# Data on Europe-wide public preferences for plankton-based ecosystem services and marine biodiversity from a series of deliberative monetary valuation workshops

**DOI:** 10.1016/j.dib.2025.111488

**Published:** 2025-03-24

**Authors:** Gilles Jean-Louis, Julian R. Massenberg, Bartosz Bartkowski

**Affiliations:** a Department of Economics, Helmholtz Centre for Environmental Research–UFZ, Permoserstraße 15, Leipzig 04318, Germany; b Department of Environmental Science–Environmental Social Science and Geography, Aarhus University, Frederiksborgvej 399, 7407, 127, Roskilde 4000, Denmark

**Keywords:** Biodiversity, Deliberative choice experiment, Discrete choice experiment, Ecosystem services, Marine ecosystems, Stated preference, Economic valuation

## Abstract

Our article describes data from a discrete choice experiment conducted within a series of deliberative monetary valuation workshops. The workshops were held across Europe and explored public preferences for plankton-based ecosystem services and marine biodiversity. In total, data were collected from 15 workshops in five different countries. In each region, workshops were carried out in both coastal and inland locations. The dataset includes responses from 172 respondents, obtained between October 2023 and February 2024. In addition to respondents’ preferences, socio-demographic data and other personal characteristics, postcodes, prior knowledge, beliefs and motivations were elicited. The data can be used to conduct custom analyses of preferences and willingness to pay for the aspects covered in the choice experiment, also incorporating the respondent-specific explanatory variables collected.

Specifications Table

**Table:** 

Subject	Behavioural Economics, Nature and Landscape Conservation.
Specific subject area	Public preferences, deliberative monetary valuation, discrete choice experiment on marine biodiversity*.*
Type of data	Comma-separated values (.CSV). Table.
Data collection	The data were collected during a series of deliberative monetary valuation workshops between 11.10.2023 and 07.02.2024. The 15 workshops were held in 5 different European regions. Prior knowledge, preferences, motivations, and beliefs were collected via questionnaires during the workshops, while socio-demographic data and information on other personal characteristics were collected beforehand as part of a screening process. The screening was designed to ensure gender balance and representativeness in terms of age, income and educational level. The sample size is n = 172.
Data source location	Poland (Poznan, Sopot) Italy (Padova, Chioggia) Basque Country / Spain (Vitoria-Gasteiz, Bilbao) Germany (Bremerhaven, Hannover) France (Rennes, Brest)
Data accessibility	Repository name: zenodo Data identification number: 10.5281/zenodo.12638010 Direct URL to data: https://doi.org/10.5281/zenodo.12638010
Related research article	*None.*

## 1 Value of the Data

• Data on public preferences and willingness to pay for marine biodiversity and associated ecosystem services are crucial for decision-makers and policy-makers to avoid potential welfare losses due to ecosystem degradation.
• The advantage of the preferences elicited by deliberative approaches is that they are based on a social preference formation process, which makes them more reliable particularly in the context of unfamiliar and complex public goods. The rich qualitative and quantitative auxiliary data allow for in-depth analyses of the determinants of the elicited preferences for marine biodiversity and ecosystem services.
• Comprehensive deliberative monetary valuation studies across countries are rare; our data allows for cross-regional analyses.
• The discrete choice experiment data can be used to examine preferences and willingness to pay for marine biodiversity and associated ecosystem services using plankton as a case study. Additionally, collected personal characteristics may be included in the analysis as well.
• Researchers may also investigate open text answers related to the discrete choice experiment.

## 2 Background

The data were collected in the context of the Horizon Europe project BIOcean5D – Marine Biodiversity Assessment and Prediction Across Spatial, Temporal and Human Scales. The aim of the study was to elicit public preferences for marine biodiversity and ecosystem services, with a particular focus on plankton. Given the importance of intangible, non-use and indirect use values in assessing biodiversity, stated preference methods, such as discrete choice experiments (DCE), are considered particularly well-suited for this purpose [1]. Moreover, it has been argued that the economic value of biodiversity is complex [2] and the usual assumptions of pre-existing preferences is unlikely to hold [3]. Therefore, allowing for social preference formation appears important [4]. Deliberative monetary valuation (DMV) approaches have been developed with such challenges in mind [5]. Accordingly, we conducted a series of deliberative choice experiments in coastal and non-coastal locations across Europe to capture public preferences for marine (plankton) biodiversity and ecosystem services.

## 3 Data Description

The dataset comprises a CSV file containing the data table and the corresponding codebook in PDF format. The data table displays the collected responses and personal characteristics for each respondent in wide format, with each row containing all the information pertinent to one individual, indicated by the respondent ID (n = 172). The data are fully anonymous and do not allow for tracing back to individuals. The variables “Country”, “Location” and “Date” indicate the geographical location and temporal context of the workshop in which the data pertaining to a specific respondent were collected. The workshop ID (“WS_ID”) and seat number (“Seat”) provide information on the specific workshop attended and the respondent's place of seating in the room where the workshop was conducted (relevant for the associated, unpublished qualitative data). The remaining variables are described in greater detail in the codebook sheet, with the exception of the information on the choice situations of the DCE ("CS"). With regard to the latter, the value assigned to each choice situation (CS) indicates the selected scenario, namely scenario one (1), scenario two (2) or the opt-out alternative (3). In the codebook, the term “Variable Label” refers to the variable names in the data set. The column labelled “Question” displays the corresponding questions and is followed by the response codes used in the data table, as well as the corresponding response labels used in the actual questionnaire. Responses in the form of open text are provided in the original language. Non-decipherable words were indicated using an “X”. The following materials are available from the Zenodo repository:

• DMV data and codebook
• Supplementary material:
  ○ Workshop guideline for facilitators in English
  ○ English version of the screening questions and workshop materials (questionnaires and a one-page overview of all DCE attributes and levels)
  ○ Original workshop materials in local language used in each workshop (questionnaires and a one-page overview of all DCE attributes and levels)
  ○ Shapefile containing the target areas for each implementation scenario
  ○ Presentations shown in each region (in local language)
  ○ Information on the experimental design of the DCE (Ngene code and final design)

## 4 Experimental Design, Materials and Methods

The survey workshop series commenced with the first workshop in Poznan on 11 October 2023 and finished with the final one on 7 February 2024 in Brest (for precise dates, please refer to the data table in the column labelled “Date”). The schedule for all workshops was identical (for details, please see Table 1 and the supplementary material in the repository).

**Table 1:** Workshop schedule of the deliberative monetary valuation workshops.

Topic	Duration
Introduction	00:00 – 00:10
Pre-Questionnaire	00:10 – 00:20
Introduction to Discrete Choice Experiment	00:20 – 00:40
Content Questions	00:40 – 00:55
Discussion	01:05 – 01:35
Post-Deliberation Discrete Choice Experiment	01:35 – 01:45
Post-Questionnaire	01:45 – 02:00

In each workshop, a professional facilitator was responsible for guiding the participants through the aforementioned procedure, utilising presentation slides (see the supplementary material in the repository). Prior to the commencement of each workshop, participants were requested to read and confirm their understanding of data privacy provisions and the study's purpose and scope. This included a disclosure that the Helmholtz Centre for Environmental Research (UFZ) was conducting the survey workshops as part of a European-wide study within the Horizon Europe project BIOcean5D. Besides its informative aspect, this disclosure was also intended to underscore the study's credibility and highlight its potential policy impact, thereby reinforcing its consequentiality. Following a brief introduction to the research project, participants were requested to complete the pre-questionnaire, which elicited attitudes, beliefs and prior knowledge. Subsequently, the facilitator provided the relevant information for completing the DCE. This was followed by an opportunity for participants to pose any remaining queries regarding the DCE. In order to ensure that participants could also pose questions regarding the marine environment, one or two marine biology experts were present at this workshop phase (these varied across locations to ensure that all discussion could take place in the local language). Prior to completing the DCE, a discussion phase was initiated by the facilitator during which attendees discussed the perceived complexity of plankton ecosystem service (ES) provision, personal vulnerability to changes in plankton diversity/composition, and conservation efforts in the face of scientific uncertainty. The objective of the discussions was to ensure preference formation, including facilitation of the adoption of a broader societal perspective. Finally, the participants were requested to complete the DCE and a follow-up questionnaire. All workshops were audio and video recorded, to which all participants had consented beforehand.

**Fig. 1:**
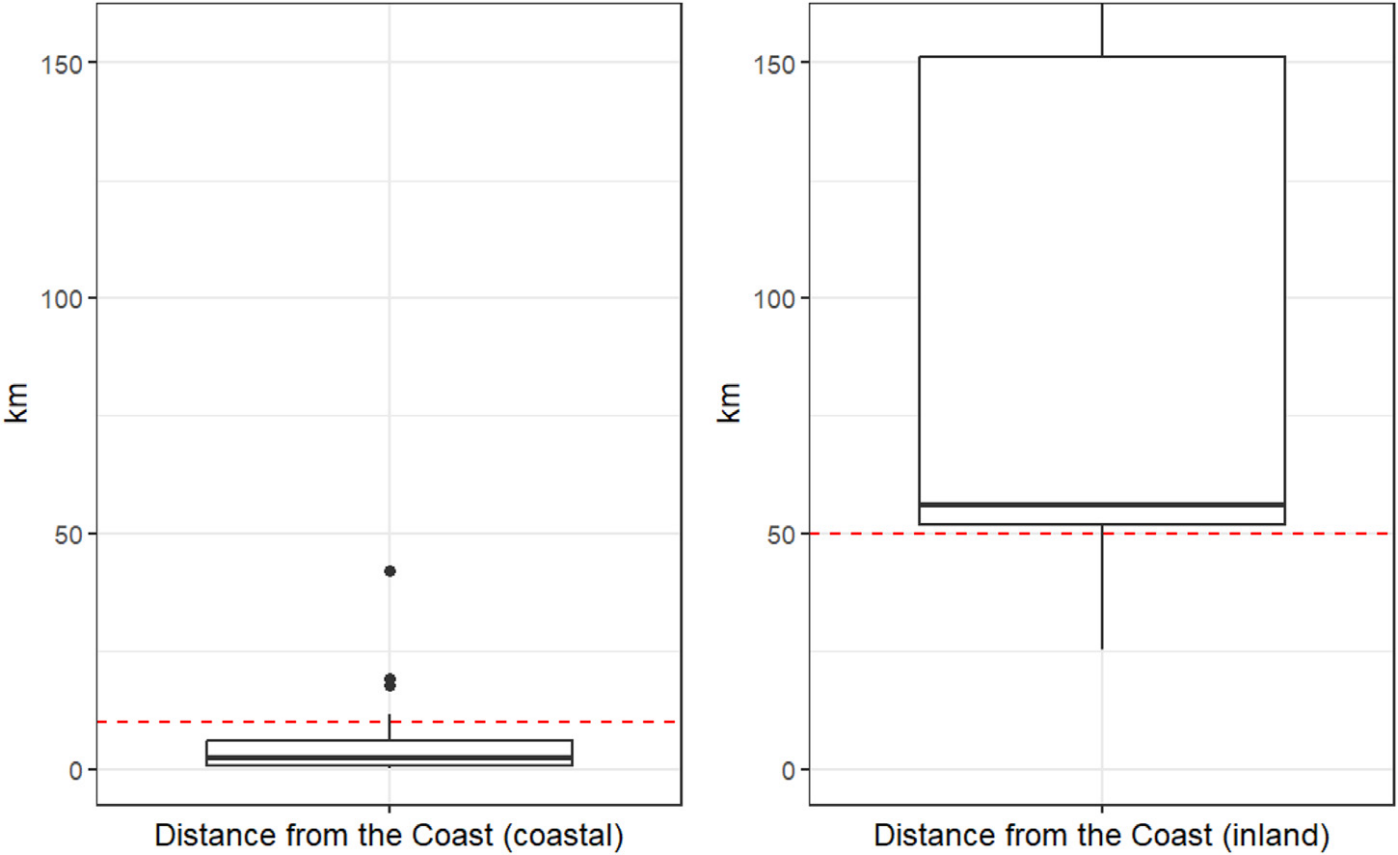
Box plots showing the distribution of the distance in kilometres from the coast to the participants’ main residence for both coastal and inland workshops attendees^1^. The red dotted lines mark the upper (coastal) and lower (inland) limits sought in the recruitment process. Of the outliers (n=32), approximately half (15 to 17) may be attributed to spatial inaccuracy associated with the postcodes, as the observed deviations were less than 2 kilometres.

The workshop participants were recruited from online panels by a market research company (Innofact AG, https://innofact-marktforschung.de/) on the basis of the screening questions provided by the research team (see “Screening Questions and Workshop Materials_EN”). The screening questions were designed to ensure broad representativeness in terms of age, income, education level and gender (Table 2), and to guarantee that only coastal and inland residents attended the respective coastal and non-coastal workshops. In the case of the coastal workshops, only lay people (i.e. no marine scientists) residing less than 10 km from the coast were to be invited, while lay participants in the inland workshops were required to live at least 50 km from the coast (Fig. 1). In addition, individuals employed in market research were excluded by default. A total of 12 participants were sought for each workshop. The actual number of workshop participants ranged between 10 and 13. The survey results comprise responses to the following questionnaire sections:

• Screening questionnaire on socio-demographic data and leisure activities
• Pre-questionnaire on attitudes, beliefs and prior knowledge
• Discrete choice experiment
• Post-questionnaire on discrete choice experiment

**Table 2:** Selected socio-demographic variables of workshop participants and comparison with the corresponding actual EU data.

Socio demographic	Sample	EU 27
% Female	51.7	51.1^1^
Age (median)	44	44.5^2^
% Educational level (ISCED 0-2)	14.5	26.7^3^
% Educational level (ISCED 3-4)	46.5	44.9^3^
% Educational level (ISCED 5-8)	37.8	29.5^3^
Monthly net income		
0 - 500 €; 0 - 2,225 PLN	10.5 %	
501 - 750 €; 2,226 - 3,338 PLN	2.3 %	
751 - 1000 €; 3,339 - 4,450 PLN	16.9 %	
1,001 - 1,250 €; 4,451 - 5,563 PLN	9.9 %	
1,251 - 1,500 €; 5,564 - 6,675 PLN	16.3 %	
1,501 - 1,750 €; 6,676 - 7,788 PLN	9.3 %	
1,751 - 2,000 €; 7,789 - 8,900 PLN	8.7 %	
2,001 - 2,250 €; 8,901 - 10,013 PLN	4.1 %	
2,251 - 2,500 €; 10,014 - 11,125 PLN	6.4 %	
2,501 - 2,750 €; 11,126 - 12,238 PLN	2.9 %	
2,751 - 3,000 €; 12,239 - 13,350 PLN	4.7 %	
> 3,001 €; > 13,351 PLN	7.6 %	
NA	0.6 %	

1 Eurostat, Population on 1 January by age and sex, (2024). https://doi.org/10.2908/DEMO_PJAN.

2 Eurostat, EU median age increased by 2.3 years since 2013 - Eurostat, (2024). https://ec.europa.eu/eurostat/de/web/products-eurostat-news/w/ddn-20240215-1 (accessed March 27, 2024).

3 Eurostat, Population by educational attainment level, sex and age (%), (2023). https://doi.org/10.2908/EDAT_LFS_9903.

Prior to the actual survey workshops, a focus group and a pre-test were conducted in English in July 2023. The objective of these preliminary studies was twofold: firstly, to ensure the comprehensibility of the entire questionnaire; secondly, to obtain preliminary results that could be used to improve the experimental design of the DCE.

### 4.1 Socio-demographic data

The screening questionnaire was employed to obtain data regarding the following characteristics of each participant:

• Gender
• Year of birth
• Educational level based on the International Standard Classification of Education [6]
• Postcode
• Number of children
• Monthly net income after tax
• Number of stays spent immediately at or on the sea in the last 12 months
• Duration of stays (if time was spent at or on the sea)
• Activities engaged in at or on the sea (if time was spent at or on the sea)

### 4.2 Pre-questionnaire

In the pre-questionnaire section, participants were inquired about their attitudes, beliefs, mindset, and prior knowledge. Initially, participants were required to self-assess their interconnection with the following entities using scale-based items:

• Nature (based on Schultz [7])
• Society (based on Mashek [8])
• Ocean

This was followed by the Environmental Portrait Value Questionnaire [9], which included 17 items in a randomised order. The remaining questions of this section of the survey focused on the respondents’ existing knowledge and awareness of marine ecosystem functions and ES. The following areas were explored:

• Knowledge about the basis of the marine food web
• Frequency of concern about the importance of marine biodiversity for personal well-being
• Self-assessed knowledge on plankton's role in the marine environment
• Inquiry about potential benefits people may obtain from the ocean and its biodiversity

### 4.3 Discrete choice experiment

The participants were required to complete a hypothetical DCE, comprising 16 distinct choice situations. In each situation, they were asked to select their preferred alternative from three potential options: two generic alternatives and a business-as-usual alternative. The data obtained from the DCE presents the alternatives chosen by each participant for each choice situation. Prior to the completion of the DCE, participants were explicitly instructed by the facilitator to respond to each choice scenario independently, neglecting previous scenarios (see “DMV Guideline for facilitators_EN” in the supplementary material). Furthermore, participants were reminded to only make selections that reflect their actual willingness to pay to encourage careful decision-making. This served to reinforce the perceived realism of the choice task and reduce hypothetical bias. The design of the DCE is described in the following section.

#### 4.3.1 Implementation scenario

**Fig. 2:**
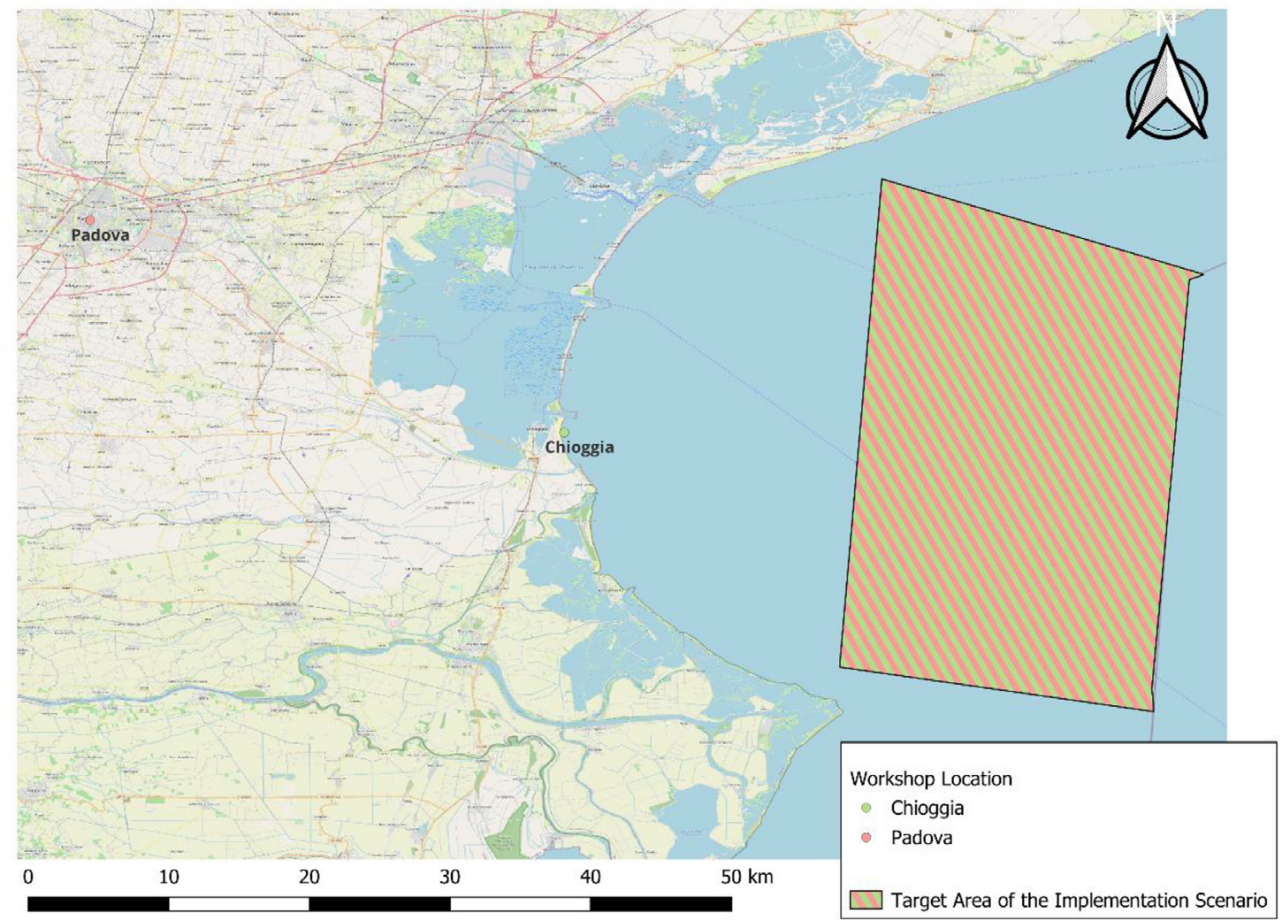
Italian workshop locations and target area of the implementation scenario.

Firstly, an implementation scenario (i.e. framework) was developed in order to elucidate the reasons for the varying hypothetical scenarios of the DCE and to specify the locations where the effects will take place. According to this implementation scenario, a mix of policies was about to be implemented by the state in order to protect marine biodiversity and increase ocean health. Starting from the nearest point within territorial waters (the reference was the place of residency) this would affect an ocean segment of 25 km in both directions within territorial waters (ca. 22 km from the coastline). In all cases, the total area affected by the measures would thus cover approximately 50 km x 22 km (≈ 1000 km²). Fig. 2 provides an illustrative example of the extent of the target area affected by the new policies for individuals residing in the city centre of the two Italian workshop locations. Please refer to the provided shapefiles and presentation slides for each workshop for further details on the target areas. It was requested that participants consider the fact that measures will affect the entire target area, rather than solely the coast, and that neighbouring regions may also be affected. The mix of policies may encompass a range of measures taking place directly in the ocean but also inland. For example, regulatory measures could be introduced to control the agricultural sector and its associated practices with the aim of reducing nutrient emissions to waterbodies. One potential policy tool that could be directly implemented in the marine environment is the designation of new Marine Protected Areas (MPA) and the expansion of existing MPAs to encompass 30% of the target area.

#### 4.3.2 Attributes and levels

**Fig. 3:**
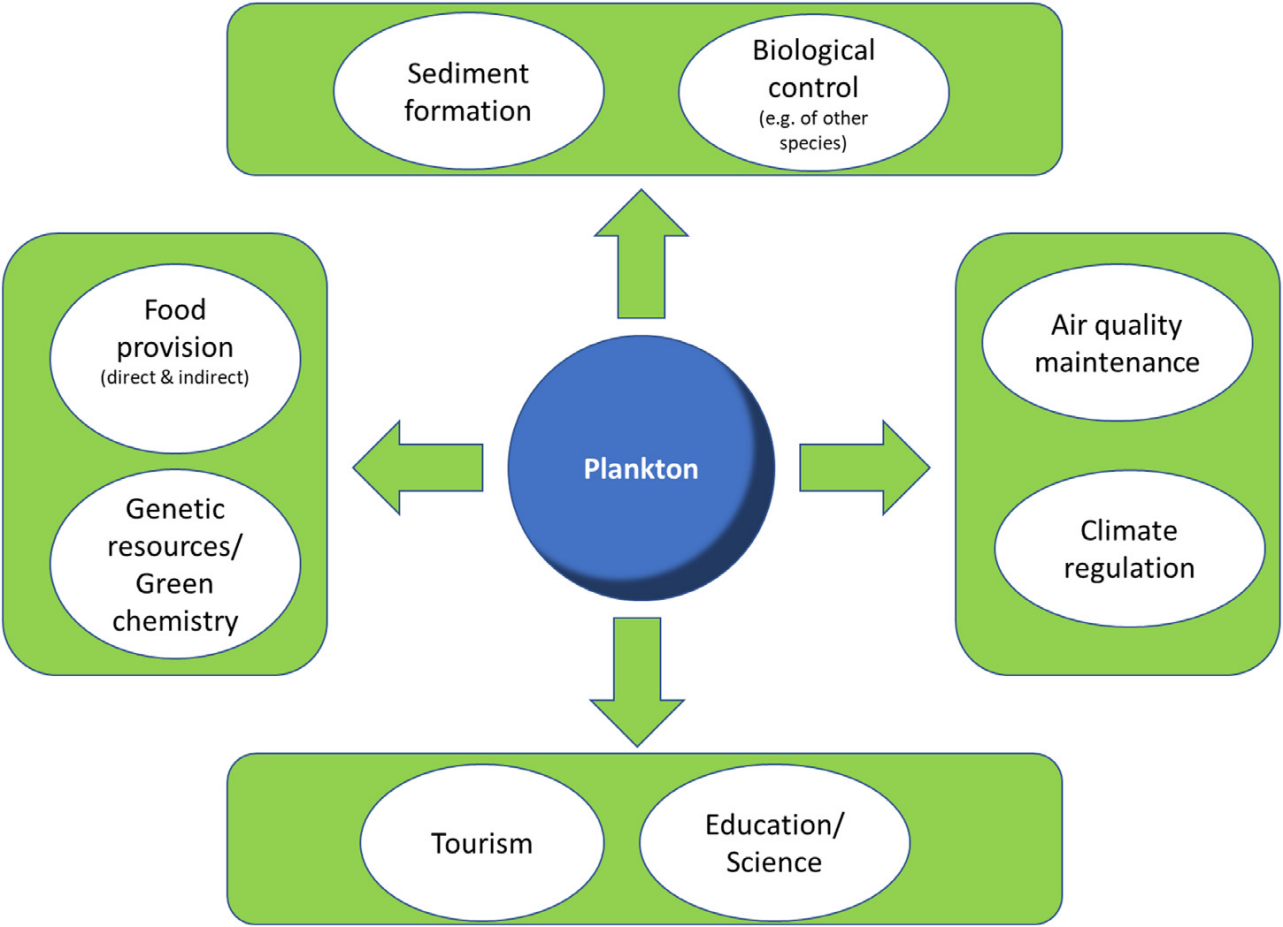
Ecosystem Services provided by (phyto-) plankton; adapted from Naselli-Flores and Padisák [10] and modified.

As the intention of the study was to elicit preferences and willingness to pay for phytoplankton and zooplankton ES, a literature review was conducted in order to identify the most relevant services. In line with the findings of Naselli-Flores and Padisák [10] and Botterell et al [11], a number of relevant ES provided by phytoplankton and zooplankton were identified and included in the study design. The most pertinent plankton-based ES are displayed in Fig. 3 and were presented to the survey participants.

The (reliability of the) provision of all plankton-based ES was covered by the attribute “plankton composition”, which could take on the levels “more stable” or “less stable”. The latter was considered to result in a decreased or unstable provision of all associated ES (e.g.: food provision, sediment formation, biological control, air quality maintenance, climate regulation, recreational fishing, tourism, genetic resources). Conversely, a more stable plankton composition was considered to ensure the continued provision of these services.

Furthermore, the regulation of algal blooms and jellyfish blooms in the target area was introduced as an attribute. The probability of both events occurring could be higher, indicating a greater likelihood of their occurrence in the specified target area than is currently observed. Thus, this attribute can be considered an ecosystem disservice or environmental public bad.

To define the climate regulation attribute, the net primary production data from Boyd et al. [12] were employed to estimate the carbon binding potential of phytoplankton in the target area. Based on the assumption that 1% of the net primary production is sequestered [13], it was estimated that an average of 1,000–3,000 t of carbon could be stored annually in the sediment of each target area. In accordance with the aforementioned status quo, the following levels were employed in the DCE: +0%, +50%, +100% or +150%.

**Fig. 4:**
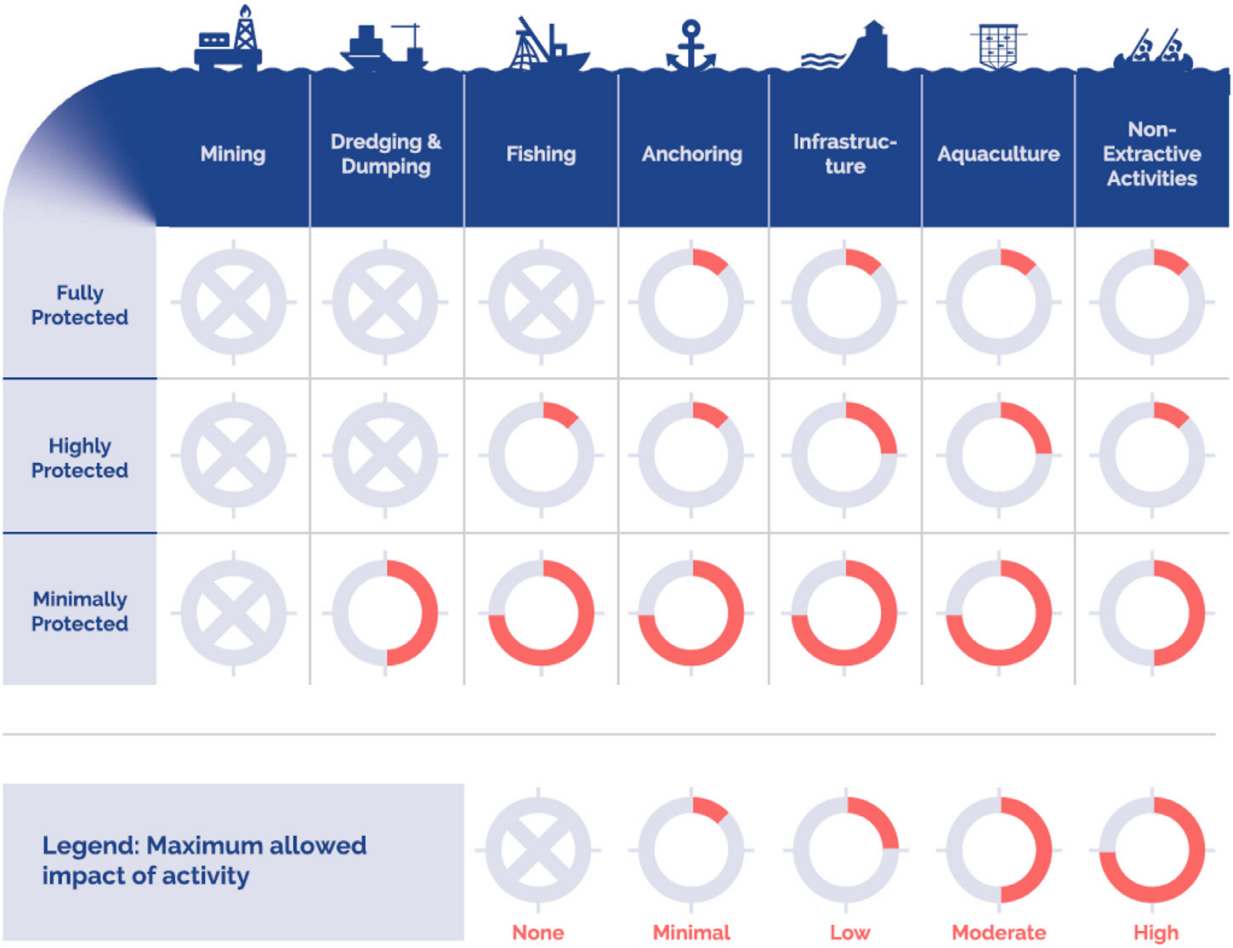
Protection levels of marine protected area taken from Grorud-Colvert [14] and modified.

In order to incorporate marine biodiversity aspects that extend beyond planktonic organisms, the protection status of an MPA was included as an attribute. This attribute served as a proxy for both conservation measures and habitat provision on the one hand, and restricted human use potential on the other. As outlined in the implementation scenario, 30% of each target area would be designated as an MPA. The level of protection attributed to this MPA would be indicated by the following attribute levels: “minimally protected” serving as the baseline, “highly protected” and “fully protected”. Definition and description of these levels were based on a review by Grorud-Colvert et al [14]: each protection level was defined by the permitted impact of certain human activities (mining, dredging & dumping, anchoring, infrastructure, aquaculture, fishing and non-extractive activities) as illustrated in Fig. 4.

In order to derive willingness-to-pay estimates for each of the non-monetary attributes, a cost attribute was included in the DCE. This was defined as an annual tax levied by the state on all adult individuals for a ten-year period to cover the costs associated with the implementation of the measures. The potential justifications for this were presented as follows: compensation for farmers for the reduction of fertiliser and operating costs for MPAs, including costs related to employment, administration and monitoring, as well as the relocation of fishing operations. It was necessary to make an adjustment to the cost attribute included in the Polish questionnaires, given that Poland is not part of the Euro zone. Accordingly, the cost levels were converted from euros (representing the average value for the European Union) to a corresponding amount of Polish złoty (PLN/zł). This was achieved by utilising currency exchange data^2^ and purchasing power parity data^3^. Following the conversion, the cost levels were rounded to the nearest ten in order to prevent the emergence of anomalous values. Table 3 presents a comprehensive overview of all DCE attributes and their corresponding levels.

**Table 3:** Attributes and corresponding levels of the discrete choice experiment.

Attribute	Description	Levels	
		Business as usual	Scenario 1 & 2
Plankton composition	A more stable plankton composition will be considered to lead to the continued provision of all plankton-based ecosystem services e.g.: food provision, sediment formation, biological control, air quality maintenance, climate regulation, recreational fishing, tourism, genetic resources whereas a less stable plankton composition is considered to lead to a decreased or unstable provision of these services	Less stable	Less stable, More stable
Higher probability of algal blooms and jellyfish blooms	Both types of blooms may occur more frequently or less frequently than at present in the target area	Yes	Yes, No
Protection status of Marine Protected Area	30% of the target area will be designated as an MPA with one of the three possible protection statuses	Minimally protected	Minimally protected, Highly/Well protected, Fully protected
Climate regulation	The carbon storage potential of the target area increases by the indicated percentage	+0%	+0%, +50%, +100%, +150%
Cost	Additional annual tax for a period of ten years that has to be paid by every citizen	0€ (0zł)	10€ (30zł), 20€ (60zł), 40€ (110zł), 80€ (230zł), 120€ (340zł), 180€ (510zł)

#### 4.3.3 Experimental design

**Fig. 5:**
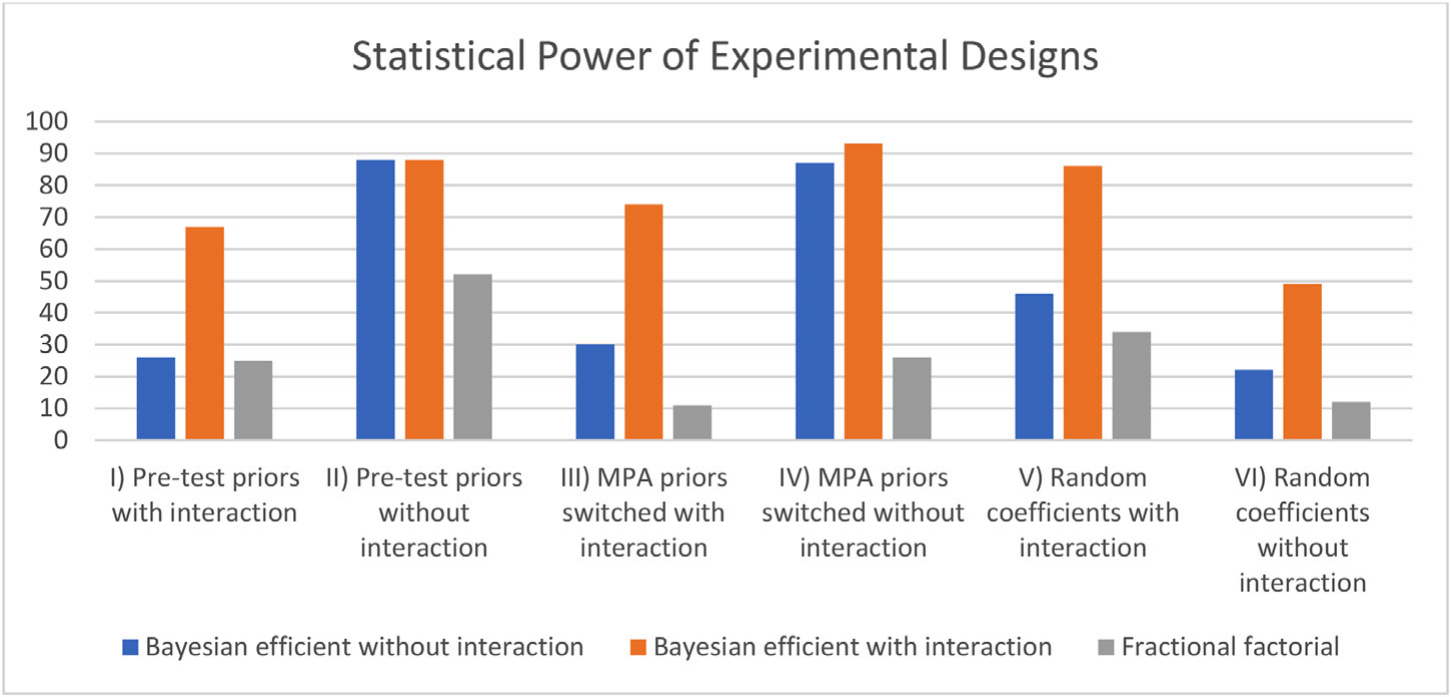
Statistical power of the tested experimental designs. The values represent the probability of detecting a statistically significant effect (assuming the presence of a true effect) for each predefined coefficient with a given experimental design on a scale from 0 to 100.

The term “experimental design” is used to describe a systematic methodology for the generation of a series of choice situations that will be presented to DCE respondents. These situations are based on a set of previously determined attributes and corresponding levels (cf. Table 3), with the objective of gathering the maximum amount of information possible about the trade-off decisions that respondents are required to make. It was deemed reasonable to assume that a higher probability of algal blooms and jellyfish blooms could only occur if the plankton composition attribute was less stable. Consequently, the two corresponding attributes were consolidated into a single attribute comprising three dummy-coded levels: “more stable plankton composition without a higher probability of algal blooms and jellyfish blooms”, “less stable plankton composition without a higher probability of algal blooms and jellyfish blooms” and “less stable plankton composition with a higher probability of algal blooms and jellyfish blooms”. For the sake of clarity, however, they were presented as two distinct attributes in the DCE. The attribute “protection status of MPA” was the second attribute to be dummy-coded (for the final experimental design), as participants may not receive a linear increase in utility from increasing protection measures. This is because such measures also imply a decrease in use for human activities. Several experimental designs were generated using the Ngene software, version 1.3.0 [15], based on the aforementioned attributes and levels. A fractional factorial design comprising 16 choice sets, randomly drawn from a full set of 46,656 possible choice sets (i.e. the full factorial design) was created initially. This design had been pre-tested in August 2023 with seven respondents. Additionally, participants in the focus group and pre-test were queried about potential fatigue effects associated with the completion of the 16 choice cards. As no indication of a fatigue effect was observed, the number of choice scenarios was maintained for subsequent experimental design generation. This was done to ensure the greatest statistical power possible, given the smaller sample size typical of a DCE in a DMV workshop format. A more sophisticated experimental design was created using coefficients (and corresponding standard errors) derived from the pre-test results as priors. To derive the pre-test coefficients to serve as priors, we employed a simple conditional logit model in preference space. Subsequently, two Bayesian D-efficient designs were generated [16], one of which included a potential interaction effect between the plankton composition attribute and the climate regulation attribute. These two designs were then compared with the initial fractional factorial design using the R package *simulateDCE* [17]. Further details regarding the Ngene code employed for the generation of the designs can be accessed in the supplementary materials. DCE results for all three experimental designs were simulated using the same predefined coefficients for each test set. A total of six sets of predefined coefficients were tested. In the initial two simulation scenarios, the pre-test coefficients were employed with (I) and without (II) an additional interaction effect in the simulation. In the third and fourth simulations, the pre-test coefficients of the dummy-coded MPA attribute levels were switched to account for a potential non-linear utility associated with this attribute. This was simulated both with (III) and without (IV) the aforementioned interaction effect. Finally, a set of randomly chosen coefficients was used to simulate the results, with (V) and without (VI) interaction between the plankton composition and the climate regulation attribute. In all six scenarios tested, the Bayesian efficient design with a priori consideration of a possible interaction effect demonstrated the best performance, with the exception of test scenario II, where the second Bayesian D-efficient design (without consideration of a priori interaction) exhibited comparable performance (refer to Fig. 5).^4^ Consequently, for the main study, we opted to use the Bayesian efficient design with a priori consideration of a possible interaction effect.

### 4.4 Post-questionnaire

The objective of the follow-up questions posed in the post-questionnaire was to gain deeper insights into the underlying motivations that shaped the participants’ preferences. The following aspects were addressed:

• General motivation
• Specific motivation related to plankton ES
• Perceived importance rating of plankton's contribution to specific ES
• Importance rating of DCE attributes
• Importance rating of several consideration statements related to scientific uncertainty, complexity and value dimensions
• Choice certainty
• Perceived difficulty related to the marine ecosystem
• Perceived future affectedness by changes in marine biodiversity
• Assessment of the scientific basis provided
• Self-assessed evaluation of the influence of other participants during the discussion
• Discussion points that influenced the choices

## Limitations

Although the sample size (n=172) is relatively large for a deliberative choice experiment, it is substantially smaller than that typically employed in conventional DCEs. Consequently, the statistical power and representativeness of the sample may be constrained. Moreover, due to content and time constraints, it was not possible to explore all potentially interesting aspects in a quantitative manner within the questionnaire. For instance, a direct question on consequentiality was not included. However, researchers interested in this or similar aspects are encouraged to analyse Q9, an open-ended follow-up question which examined respondents' motivations for choosing specific choice scenarios, using feasible indicators of consequentiality. As previously stated, participants were required to engage in group discussions on specific topics in order to facilitate a more societal perspective. However, this approach also carries the potential risk of introducing bias. As different facilitators and experts were employed in each region, there is a possibility of bias, despite the fact that all facilitators followed the same guideline and presented the same materials. On the other hand, DMV rests on the assumption that preference formation is an inherently social process, which can never be perfectly controlled by the researcher.

## Ethics Statement

Prior to participation in one of the survey workshops, the workshop participants were required to provide informed consent. The requisite forms and the study design have been approved by the Ethics Committee of Martin-Luther-Universität Halle-Wittenberg (Ref.Nr.: 2309JGE). Accordingly, no ethical concerns were identified.

## Funding

This work was funded by the European Union in the framework of the Horizon Europe project “BIOcean5D” (GA#101059915). The views and opinions expressed are those of the author(s) only and do not necessarily reflect those of the European Union. Neither the European Union nor the granting authority can be held responsible for them.

## CRediT authorship contribution statement

**Gilles Jean-Louis:** Conceptualization, Methodology, Data curation, Investigation, Formal analysis, Visualization, Writing – original draft, Writing – review & editing. **Julian R. Massenberg:** Conceptualization, Methodology, Funding acquisition, Writing – review & editing. **Bartosz Bartkowski:** Conceptualization, Methodology, Funding acquisition, Writing – review & editing, Supervision.

## Data Availability

ZenodoEurope-wide public preferences for plankton-based ecosystem services and marine biodiversity from a series of Deliberative Monetary Valuation workshops (Original data).

## Footnotes

1 The distance from the coast was calculated post hoc based on the provided postcodes, corresponding centroids (https://ec.europa.eu/eurostat/web/gisco/geodata/administrative-units/postal-codes), and spatial data on the European coastline (https://www.eea.europa.eu/data-and-maps/data/eea-coastline-for-analysis-1/gis-data/europe-coastline-shapefile).

## Footnotes

2 European Central Bank, Euro foreign exchange reference rates - Polish zloty (PLN), (2023). https://www.ecb.europa.eu/stats/policy_and_exchange_rates/euro_reference_exchange_rates/html/eurofxref-graph-pln.en.html (accessed September 26, 2023).

3 Eurostat, Purchasing power parities - Comparative price levels, (2024). https://doi.org/10.2908/tec00120.

4 More detailed information on the test input and output parameters can be found in the supplementary materials (see folder “simulateDCE”).
